# Global Advances in Ventricular Tachycardia Ablation

**DOI:** 10.19102/icrm.2026.17092

**Published:** 2026-09-15

**Authors:** Fahd Mohamed, Amar Ghaleb, Abimbola Kolawole, Arfaat M. Khan

**Affiliations:** 1 Central Michigan University College of Medicine, Mount Pleasant, MI, USA; 2 Cardiology, Henry Ford Health, Detroit, MI, USA

**Keywords:** Arrhythmia management, catheter ablation, electrophysiology, myocardial scar, ventricular tachycardia ablation

## Abstract

Ventricular tachycardia (VT) is a potentially life-threatening arrhythmia associated with substantial morbidity and mortality, particularly in patients with structural heart disease. Over the past several decades, the management of VT has evolved significantly with the development of catheter-based and noninvasive ablation strategies aimed at eliminating arrhythmogenic myocardial substrates. This review provides a comprehensive overview of global advances in VT ablation, highlighting historical foundations, current therapeutic modalities, and emerging technologies shaping the future of ventricular arrhythmia (VA) management. Radiofrequency ablation remains the cornerstone of VT ablation and has undergone substantial technological refinement through innovations such as irrigated-tip catheters, high-density electroanatomic mapping, contact force sensing, and strategies for achieving deeper myocardial lesions. Alternative catheter-based energy modalities, including cryoablation and the emerging nonthermal technique of pulsed field ablation, provide additional therapeutic options with distinct mechanisms of action and safety profiles. In parallel, procedural advancements such as remote magnetic navigation have improved catheter stability and access to complex ventricular substrates. Beyond traditional catheter ablation, the therapeutic landscape has expanded to include neuromodulatory approaches targeting the cardiac autonomic nervous system, including thoracic epidural anesthesia, stellate ganglion blockade, cardiac sympathetic denervation, and renal denervation. These strategies play an important role in the management of refractory VAs and electrical storm. Furthermore, stereotactic body radiation therapy has emerged as a promising noninvasive treatment for patients with VT who are not candidates for or have failed catheter-based interventions. Additional investigational technologies including laser ablation, microwave ablation, and high-intensity focused ultrasound have been explored in preclinical and limited clinical settings but remain experimental in the context of VT management. Collectively, these evolving technologies underscore the increasingly multimodal approach required to treat complex VAs. In conclusion, VT ablation has progressed from early surgical and direct-current approaches to a sophisticated field incorporating advanced catheter techniques, neuromodulation, robotic navigation, and noninvasive therapies. Continued technological innovation, improved substrate characterization, and integration of emerging tools such as artificial intelligence are expected to further enhance procedural efficacy and patient outcomes. Individualized treatment strategies tailored to patient-specific arrhythmogenic substrates and clinical characteristics remain central to optimizing the management of VT.

## Background

The treatment landscape for cardiac arrhythmias has un-dergone a remarkable evolution over the centuries, driven by the pursuit of more effective and safer therapeutic inter-ventions. Ablation techniques, characterized by the targeted destruction of abnormal cardiac tissue responsible for erra-tic electrical signals, have emerged as pivotal approaches in the management of these conditions.

While the conceptual roots of energy-based tissue ablation trace back to early explorations with high-frequency currents, the history of ventricular tachycardia (VT) ablation as a clinical discipline began in earnest with the electrophysiological work of the late 20^th^ century. Dr. Hein Wellens and colleagues^1^ laid critical groundwork in understanding ventricular arrhythmia (VA) mechanisms, setting the stage for the development of catheter-based and surgical interventions. Equally foundational were the seminal contributions of Josephson and colleagues,^2^ whose landmark studies in the late 1970s elucidated the re-entrant mechanism of VT in the setting of prior myocardial infarction. Their meticulous endocardial catheter mapping techniques and surgical approaches to scar-related VT established the conceptual and technical framework upon which modern substrate-based catheter ablation is built, and these contributions remain among the most influential in the history of VT management **(Figure 1)**.

**Figure 1:**
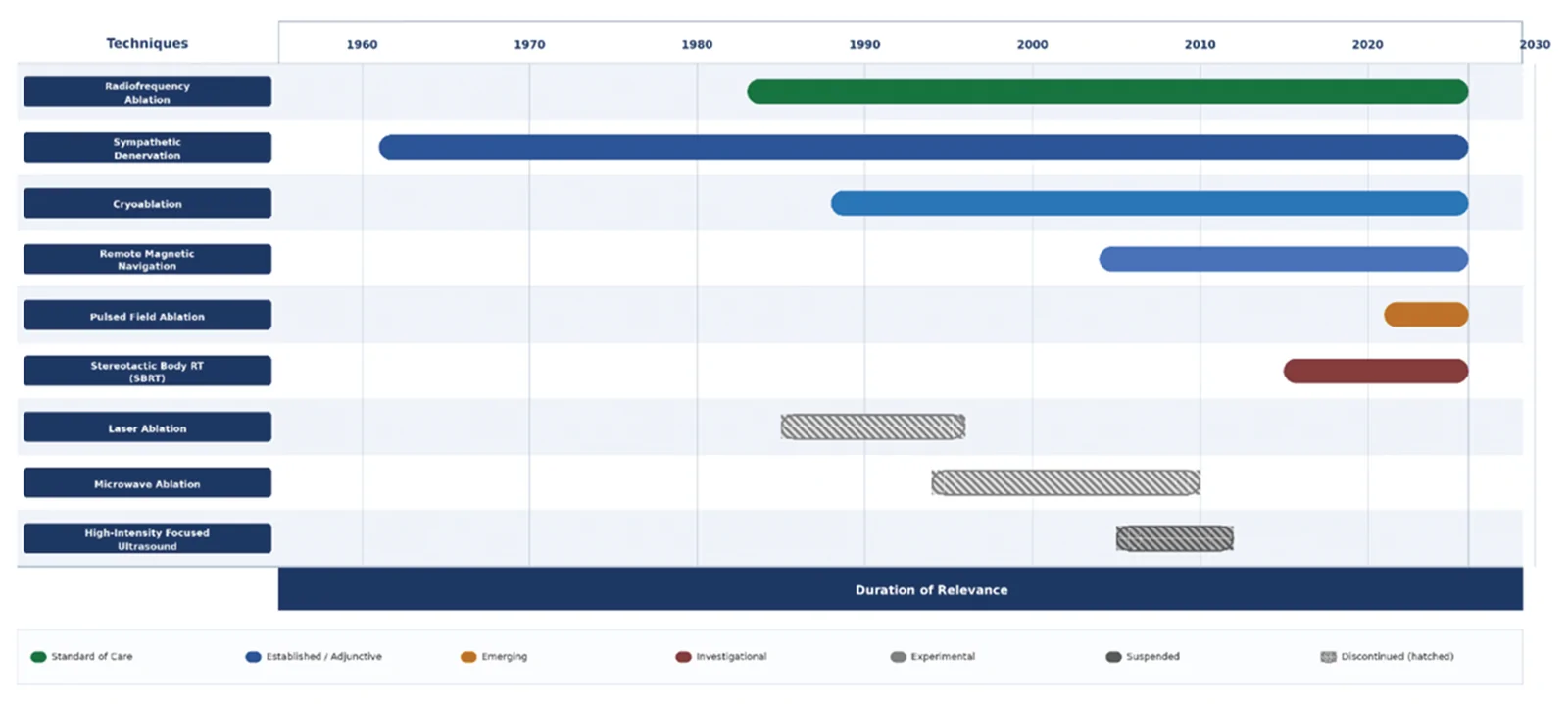
Historical and contemporary timeline of ventricular tachycardia (VT) ablation techniques. This timeline illustrates the approximate periods of clinical relevance for major VT ablation and adjunctive therapeutic techniques. Colors denote the current clinical status: green (standard of care), blue (established/adjunctive), amber (emerging), dark red (investigational), and gray (experimental or suspended). The hatched bars indicate suspended or discontinued modalities. Sympathetic denervation encompasses surgical cardiac sympathetic denervation (1961–present), thoracic epidural anesthesia and stellate ganglion blockade (1980s–present), and catheter-based renal sympathetic denervation (2010s–present). The faint vertical marker denotes 2026 (current year). *Abbreviation:* RT, radiation therapy.

Radiofrequency (RF) ablation (RFA), employing high-frequency alternating current to create thermal lesions on targeted tissue, quickly emerged as a cornerstone in arrhythmia management **(Table 1)**. Its versatility allows for the treatment of various arrhythmias, including VT, offering clinicians a powerful tool to address complex cardiac conditions. However, while RFA is highly effective, it carries risks such as thromboembolic events, tissue overheating, and potential damage to surrounding structures. The procedural success rates and complication rates can vary, necessitating careful patient selection and technological assistance.

**Table 1:** A Comparative Overview of VT Ablation Modalities

Modality	Mechanism	Main Complications	Clinical Status	Evidence Maturity
RFA	Resistive and conductive heating via high-frequency alternating current → coagulative necrosis	Cardiac tamponade, thromboembolism, phrenic nerve/esophageal injury, steam pops	**Standard of care**	High—decades of prospective data
Cryoablation	Extreme cold induces direct cellular injury and indirect cell death via unfavorable microenvironment	Smaller lesion diameter vs. RF ablation, slower lesion maturation, limited effectiveness in deep intramural or scar-related substrates, reduced catheter maneuverability	**Established (selected cases)**	Moderate—limited VT-specific RCT data
PFA	Irreversible electroporation via ultra-short high-amplitude pulsed electric fields → nonthermal cell death	Arrhythmias, coronary artery injury, hemoglobinuria/AKI, catheter maneuverability limitations	**Emerging**	Low-moderate—early stage, no VT-specific RCTs yet
RMN	Externally applied magnetic fields steer soft atraumatic catheters to precise intracardiac targets	Limited; reduced perforation risk vs. manual; operator radiation reduction	**Established (adjunctive)**	Moderate—multicenter data available, RCT (MAGNETIC VT) ongoing
Sympathetic denervation (TEA/SGB/CSD/RDN)	Blockade of cardiac sympathetic efferents via neuraxial anesthesia, ganglion excision, or catheter-based renal nerve ablation	TEA/SGB: transient; CSD: surgical risks, Horner syndrome; RDN: renal artery injury (rare)	**Established (adjunctive)**	Moderate—strongest for CSD/SGB, RDN requires further trials
SBRT	Non-invasive focused radiation → myocardial fibrosis, autonomic denervation, ion channel remodeling	Late radiation cardiac toxicity (under investigation), esophageal injury risk	**Investigational**	Low-moderate—promising early data, awaiting RCT results
Laser ablation	Photon energy → tissue absorption → coagulative necrosis via controlled thermal elevation	Specialized equipment required, cost, limited depth data in ventricular tissue	**Experimental (VT)**	Very low—no human VT evidence
MWA	Dipole rotation of water molecules via electromagnetic fields → uniform tissue heating	Depth/uniformity concerns, no ongoing device development	**Experimental (VT)**	Very low—no VT evidence, fallen out of favor
HIFU	Focused ultrasonic waves → acoustic cavitation and hyperthermia → cell death	Lethal atrio-esophageal fistula, esophageal injury, phrenic nerve palsy	**Suspended**	Very low—trials suspended, safety unresolved

*Abbreviations:* AF, atrial fibrillation; AKI, acute kidney injury; CSD, cardiac sympathetic denervation; HIFU, high-intensity focused ultrasound; ICD, implantable cardioverter-defibrillator; MAGNETIC VT, Comparison of VT Ablation Outcomes Using Remote MAGNETIC Navigation Versus Manual Approach in a Low-LVEF Population; MWA, microwave ablation; PFA, pulsed field ablation; RCT, randomized controlled trial; RDN, renal denervation; RF, radiofrequency; RFA, radiofrequency ablation; RMN, remote magnetic navigation; SBRT, stereotactic body radiation therapy; SGB, stellate ganglion blockade; TEA, thoracic epidural anesthesia; VA, ventricular arrhythmia; VT, ventricular tachycardia. This table summarizes the mechanism of action, VT-specific clinical evidence, primary complications, current clinical status, and evidence maturity for each major ablation and adjunctive modality discussed in this review. Modalities are ordered by clinical establishment, from the current standard of care to suspended or experimental techniques. Clinical status reflects the strength and maturity of available VT-specific evidence rather than general cardiac applications. Evidence maturity designations (high, moderate, low-moderate, very low) are based on the availability of RCT data, prospective multicenter studies, and long-term follow-up specific to VA management. Clinical status key: standard of care (green) = established first-line therapy; established/adjunctive (blue) = validated in clinical practice with defined role; emerging (amber) = growing evidence base, not yet standard; investigational (dark red) = active trials, not yet recommended for routine use; experimental (gray) = preclinical or minimal human data; suspended (dark red) = clinical trials halted due to safety concerns.

Cryoablation uses extreme cold to destroy abnormal tissue, offering reversible lesion creation and real-time monitoring capabilities, presenting a compelling alternative to RFA. This technique is particularly advantageous due to its ability to create well-demarcated lesions while minimizing collateral damage. The main advantages include reduced risk of thrombus formation and the ability to test lesion lines before permanent ablation (cryo-mapping). Common complications include phrenic nerve paralysis and pulmonary vein stenosis.

Pulsed field ablation (PFA) has emerged as a novel and rapidly growing modality employing irreversible electroporation to achieve nonthermal myocardial lesion formation, offering a favorable safety profile compared to thermal techniques. Several other energy-based modalities—including laser ablation, microwave ablation (MWA), and high-intensity focused ultrasound (HIFU)—have been explored in preclinical and limited clinical settings; these are addressed in a dedicated subsection on experimental and investigational approaches.

As the field progresses, future directions in ablation techniques focus on refining procedural efficiency, enhancing lesion visualization, and improving patient outcomes. Integration of advanced imaging modalities, navigation technologies, and novel therapeutic platforms holds the promise of further optimizing treatment strategies and advancing the field of cardiac electrophysiology.

## Radiofrequency ablation

### Mechanism of action

Catheter ablation encompasses various techniques employed to treat arrhythmias by targeting and ablating abnormal cardiac tissue responsible for the aberrant electrical signals. Among these techniques, RFA stands as the most prevalent, using RF energy to create thermal lesions on the targeted tissue. RFA is currently, and has been since the early 1990s, the mainstay technique for VT. Prior to the advent of RF usage to terminate VT, applying high-energy intracardiac direct-current shocks was the preferred method up till 1983. However, given the concerns about barotrauma and the need for general anesthesia with direct-current shocks, RFA was adopted by the end of the 1980s and has since become the standard ablation modality.

RF current is the principal method of ablative energy in managing cardiac arrhythmias, with its origins in electrosurgery. Over decades, RFA has evolved significantly, allowing precise treatment of various arrhythmias through high-frequency alternating current. This current generates heat within targeted cardiac tissues, leading to the formation of discrete lesions by causing controlled cell death that interrupts the electrical pathways responsible for arrhythmias. The process involves both resistive and conductive heating—resistive heating creates the primary lesion, while conductive heating extends the thermal effect to adjacent tissue, contributing to lesion growth; the resultant tissue destruction may interrupt the abnormal electrical circuits responsible for arrhythmogenesis.

Recent advancements in RFA technology have enhanced its efficacy and safety, characterized by innovations in catheter design, energy delivery systems, and real-time monitoring technologies. These developments ensure precise lesion placement and minimize the risk of unintended damage to cardiac or adjacent tissues. Initially, RFA was documented as a means for atrioventricular bypass tract ablation among patients with Wolff–Parkinson–White syndrome. By the early 1990s, RFA was being used to treat patients with both idiopathic and ischemic VT.

### Ablation techniques

RFA techniques for VT have evolved significantly, aiming to enhance procedural efficacy and safety. These techniques involve the use of specialized catheters equipped with advanced features such as optimized tip configurations and irrigation systems to improve tissue contact and energy delivery. Innovations in mapping technologies, including high-density mapping and image integration, enable precise localization and characterization of arrhythmogenic substrates, facilitating targeted ablation. Real-time monitoring tools such as contact force sensing and tissue impedance monitoring refine lesion formation, ensuring adequate tissue destruction while minimizing collateral damage.

Notable adverse risks of RFA procedures include cardiac tamponade, which may result from perforation during the procedure, and vascular complications such as embolism or bleeding. There is also the risk of unintentional damage to structures adjacent to the heart such as the phrenic nerve or esophagus. Moreover, thromboembolism can lead to serious systemic effects if clots migrate to vital organs. Advanced techniques such as focal impulse and rotor modulation and substrate-based strategies continue to refine the safety and effectiveness of RFA, providing tailored solutions for complex VT substrates.

### Clinical applications, outcomes, and safety profiles

Numerous clinical studies have demonstrated the efficacy of RFA in achieving durable arrhythmia suppression and improving quality of life in patients with VT. Procedural success rates and complication profiles vary based on patient characteristics, arrhythmia substrate, and operator expertise. Common complications of RFA include cardiac perforation, thromboembolic events, and vascular access–related complications, emphasizing the importance of meticulous procedural planning and vigilant post-procedural care.

Among patients without structural heart disease, treatment of monomorphic VT has been shown to have success rates of >95% with no reported recurrence on long-term follow-up. In a study conducted by Stevenson et al.,^3^ among 231 patients with recurrent episodes of monomorphic VT caused by prior myocardial infarction, 113 (49%) were free of inducible monomorphic VT, and, after a 6-month follow-up, 123 (53%) patients were free of recurrent incessant VT or intermittent VT. In a study conducted by Morady et al.,^4^ among 15 patients with VT and coronary artery disease, 11 (73%) were cured using several applications of RF energy with no reportable recurrences during a mean follow-up of 9.1 ± 3.3 months.

A landmark contribution to the field is SMASH-VT (“Substrate Mapping and Ablation in Sinus Rhythm to Halt Ventricular Tachycardia”), a randomized controlled trial^5^ that evaluated prophylactic catheter ablation in survivors of myocardial infarction with implantable cardioverter-defibrillators (ICDs). The trial demonstrated that substrate-based ablation performed in sinus rhythm significantly reduced the burden of appropriate ICD therapies compared to medical management alone, without increasing mortality. The SMASH-VT trial established an important paradigm for preventive VT ablation and remains a key reference in the guideline-directed management of post–myocardial infarction patients at risk of VT.

### Global advancements and future directions

There have been many notable studies addressing advancements made toward enhancing the efficacy, user practicality, and safety profile of RFA. Safely achieving a deeper lesion has been among the most heavily investigated challenges, given studies demonstrating improved ablation efficacy in ischemic VT from larger lesions. External irrigation with normal saline in RFA reduces lesion size compared to half-normal saline and dextrose, which create larger lesions. Impedance modulation through additional electrode patches and low-ionic irrigants with high power can increase lesion size, albeit with an elevated risk of complications such as steam pops and strokes. Employing two catheters in simultaneous unipolar or bipolar configurations has also shown promising results in achieving deeper lesions, demonstrating success in small series for VT termination where sequential unipolar ablation failed. However, these techniques require larger studies to confirm safety.

A particularly innovative approach to deep substrate ablation has been the development of needle ablation catheters by Stevenson et al., enabling the delivery of RF energy directly into the myocardium via an extendable needle electrode. This technique addresses one of the most persistent limitations of conventional catheter ablation—the inability to create lesions of sufficient depth to reach deeply intramural or epicardially inaccessible VT circuits. In selected patients with refractory scar-related VT at specialized centers, needle ablation has demonstrated the ability to eliminate arrhythmia substrates that proved unresponsive to conventional endocardial and epicardial approaches. While currently limited to a small number of expert centers, this technique represents an important frontier in substrate-based VT therapy.

Emerging technologies such as contact force sensing, high-resolution mapping, and artificial intelligence–driven algorithms hold promise in optimizing lesion quality, procedural efficiency, and long-term outcomes. Recent developments in ablation technology for VT have focused on enhancing the efficacy of RF energy delivery to the myocardium, introducing innovative agents to modify the ohmic resistance of the ablation circuit, and exploring therapeutic strategies for areas previously inaccessible using conventional techniques. Ongoing research efforts aim to expand the indications for RFA to encompass novel arrhythmia substrates and patient populations, paving the way for the continued evolution of cardiac electrophysiology.

## Remote navigation systems in ventricular tachycardia ablation

Remote magnetic navigation (RMN) systems, such as the Niobe platform (Stereotaxis, Inc., St. Louis, MO, USA), allow operators to steer soft, atraumatic catheters within cardiac chambers using externally applied magnetic fields controlled from outside the fluoroscopy suite. Rather than relying on manual catheter torque and push–pull mechanics, RMN achieves precise catheter tip orientation by varying the direction and magnitude of external magnetic fields, enabling navigation to anatomical targets that may be difficult or impossible to reach reliably with conventional catheters.

In the context of VT ablation, RMN has been evaluated in several clinical series and registry-based studies. Outcomes have been generally comparable to those achieved with conventional manual ablation, with some series reporting favorable procedural success and complication rates. The proposed advantages of RMN include enhanced catheter tip stability at the target site; a reduced likelihood of cardiac perforation due to the passive compliance of the catheter; decreased operator radiation exposure (as the operator controls the system from outside the fluoroscopy field); and improved accessibility to challenging anatomical regions, including the interventricular septum and apical segments.

RMN-guided ablation may be particularly relevant for patients with structurally complex VT substrates, including those with nonischemic cardiomyopathy, where arrhythmogenic tissue can be distributed in locations difficult to access with standard manual catheters. While RMN has not supplanted conventional catheter techniques as the standard of care for VT ablation, the accumulated clinical experience and published literature support its inclusion as an established adjunctive navigation technology in the VT ablation armamentarium. Ongoing integration of RMN with advanced electroanatomic mapping systems continues to refine its utility.

## Cryoablation

### Mechanism of action

Cryoablation is a process that entails extracting heat, leading to the destruction of tissue. It brings about tissue damage through both direct and indirect means: the direct mechanism causes injury to cells, while the indirect mechanisms create an environment unfavorable for cell survival. Cryotherapy presents distinct benefits compared to RFA, such as the capacity to create a reversible lesion by adjusting temperature prior to reaching lethal levels, making it useful for cryo-mapping. Cryoablation has emerged as a promising technique for the treatment of cardiac arrhythmias, offering an alternative to traditional RFA with distinct advantages and unique challenges.

### Clinical applications, outcomes, and safety profiles

Cryoablation procedures require meticulous attention to technical aspects, including catheter positioning, balloon inflation, and freeze–thaw cycle optimization. Cryoablation is generally recommended for the management of supraventricular tachycardias in non–atrial fibrillation cases. Cryoballoon-based systems have emerged as a popular choice for pulmonary vein isolation, offering simplicity, reproducibility, and favorable clinical outcomes compared to traditional point-by-point techniques. Cryoablation also plays a role in the treatment of VT, targeting critical isthmuses or arrhythmogenic foci within the ventricles to achieve arrhythmia suppression. The primary advantage of cryoablation over other thermal ablation techniques is the ability to monitor the ablation zone during the procedure in real time. Common complications associated with cryoablation include phrenic nerve injury, pulmonary vein stenosis, and atrio-esophageal fistula formation.

In a study by Cáceres et al.,^6^ the efficacy of cryosurgery for treating refractory monomorphic sustained VT related to inferior wall infarction was evaluated in 15 patients. Applying a mean of 9.2 ± 1.8 cryolesions per heart, the study eliminated inducible VT in 11 patients, reporting a clinical success rate of 93%, with 13 of 14 patients experiencing no late deaths, recurrence of sustained VT, or significant mitral regurgitation during a mean follow-up of 19 ± 7 months.

Recent studies have shown cryoablation to be relatively inferior to RFA for VAs. The lesions produced by focal cryoablation are typically smaller and take longer to develop compared to those created by RF ablation. Cryoablation has shown advantages in specific scenarios, such as VAs originating from highly mobile papillary muscles, where catheter adherence to the tissue during lesion creation can be beneficial. Unless further innovations improve the ability to deliver larger cryoablation lesions, the use of cryoablation for VAs will likely remain limited to specialized circumstances and as a potential adjunctive therapy.

### Global advancements and future directions

Recent advancements in cryoablation technology have focused on improving procedural efficiency, lesion visualization, and patient outcomes. Novel catheter designs, cryoballoon modifications, and imaging modalities such as intracardiac echocardiography and three-dimensional mapping systems have enhanced lesion delivery and efficacy. Globally, adoption rates of cryoablation vary, influenced by regional access to health care technology and infrastructure. Cost factors significantly affect the choice of ablation techniques, as cryoablation is generally more costly than other methods, which impacts its adoption in cost-sensitive health care settings. Future directions in cryoablation research include the exploration of adjunctive therapies, personalized procedural planning, and the integration of artificial intelligence–driven algorithms to optimize treatment strategies and improve long-term success rates.

## Pulsed field ablation

### Mechanism of action

PFA operates through irreversible electroporation, a process induced by ultra-short, high-amplitude pulsed electric fields. These fields create nanopores in cell membranes, disrupting cellular homeostasis and triggering cell death via apoptosis or necrosis. Unlike traditional thermal-based ablation methods, PFA’s nonthermal approach minimizes the risk of collateral damage to surrounding tissues, enhancing its safety and efficacy in treating cardiac arrhythmias.

### Ablation techniques

PFA’s technique involves delivering precisely controlled electrical pulses to targeted cardiac tissue using specialized catheters. These catheters are equipped with multiple electrodes positioned strategically to achieve optimal tissue contact and energy delivery. By administering ultra-short pulses, PFA ensures efficient tissue ablation while minimizing thermal effects on adjacent structures, such as the esophagus and phrenic nerve. PFA has emerged as a novel and promising method for cardiac arrhythmia ablation, offering superior safety and efficacy by minimizing collateral tissue damage while maintaining effective myocardial ablation.

### Clinical applications, outcomes, and safety profiles

PFA is an emerging technique in the field of cardiac electrophysiology, showing promise for the treatment of VT. Traditional RFA can struggle to form deep lesions in areas of scar tissue, a common challenge in VT treatment. PFA, with its nonthermal mechanism of myocardial lesion formation, offers a potential advantage by effectively penetrating both healthy and scarred tissues. Unlike RFA, PFA achieves tissue destruction through irreversible electroporation, using transient electrical discharges to induce cell apoptosis without generating excessive heat. This nonthermal approach could circumvent the limitations associated with thermal ablation techniques.

A comprehensive analysis by Zhang et al.^7^ reviewed 10 patients from eight publications, including seven men and three women, with an average age of 58.5 ± 14.2 years and a mean follow-up duration of 3.1 ± 2.0 months. The study population had a high prevalence of comorbidities, including heart failure (30%) and ischemic cardiomyopathy (50%). Notably, 70% of the patients had experienced electrical storms, and most had been treated with amiodarone and β-blockers. Despite 60% of the patients having undergone prior RF ablation with postoperative VT recurrence, all patients underwent successful procedures following standardized PFA protocols.

While PFA shows favorable outcomes, it is not without risks. Potential complications include arrhythmias, coronary artery injury, thrombus formation, and acute kidney injury due to hemoglobinuria. Some operators have also reported limitations in catheter maneuverability within the left ventricle, occasionally leading to misplacement.

### Global advancements and future directions

PFA continues to emerge as a promising technology for the treatment of VT ablation. As research progresses, numerous advancements aim to enhance its efficacy and address existing limitations. Miniaturization of PFA catheters facilitates their application in smaller and more intricate anatomical structures, enabling targeted and precise ablation. Efforts to optimize energy delivery parameters seek to achieve more efficient and uniform tissue ablation, thereby reducing the necessity for multiple applications and enhancing procedural efficiency. The integration of artificial intelligence and machine learning algorithms holds promise in augmenting the accuracy of PFA procedures by analyzing imaging data to identify optimal ablation targets. Further long-term efficacy studies are warranted as this modality continues to evolve.

## Sympathetic denervation

VAs pose a significant risk of morbidity and mortality, and understanding the role of the cardiac autonomic nervous system in their pathogenesis has led to the development of neuromodulation therapies. The sympathetic nervous system plays a crucial role in VAs by disrupting the balance between sympathetic and parasympathetic signals transmitted to the heart. Sympathetic activation can lead to the release of norepinephrine and other co-transmitters, contributing to arrhythmogenesis and heart failure. In patients with heart disease, sympathetic hyperactivity exacerbates myocardial injury, scar formation, and neural remodeling, creating an environment conducive to VT and fibrillation.

Sympathetic neuromodulation techniques—including thoracic epidural anesthesia (TEA), stellate ganglion blockade (SGB), cardiac sympathetic denervation (CSD), and renal sympathetic denervation (RDN)—have shown promise in treating refractory VAs that are unresponsive to traditional medical therapy or catheter ablation. These interventions aim to modulate sympathetic activity to reduce the risk of recurrent VAs, particularly in cases related to scar tissue.

### Thoracic epidural anesthesia

TEA is a minimally invasive therapeutic modality used for the immediate management of electrical storm in patients with VAs. This approach involves the injection of anesthetic agents into the thoracic epidural space, resulting in the inhibition of sympathetic signals to the heart. By blocking the C8 nerve roots and T1–T4 segments, which contain cardioaccelerator fibers responsible for heart rate and contractility control, TEA can provide complete sympathetic blockade. Studies have shown promising results with TEA in reducing arrhythmia burden in patients with refractory VAs, including those with ischemic and nonischemic cardiomyopathy. TEA serves as a valuable temporary intervention, stabilizing patients while more definitive treatments such as catheter ablation or advanced heart failure therapies are considered.

### Stellate ganglion blockade

SGB, performed by injecting a local anesthetic into the stellate ganglion percutaneously, is another bedside therapy that inhibits sympathetic signals to and from the heart. This method, first introduced in 1934 for treating chronic pain conditions,^8^ disrupts both incoming and outgoing sympathetic signals to and from the heart at the stellate ganglion level. The procedure can be safely carried out using ultrasound guidance alone or a combination of fluoroscopy and ultrasound. Unlike TEA, where a catheter is left in place, percutaneous SGB procedures involve repeated injections as needed and can be performed in patients on anticoagulant or antiplatelet therapy.

SGB has led to a significant reduction in VA episodes and defibrillation events within 24 and 48 h after the procedure. TEA and SGB offer the chance to stabilize the patient while addressing underlying triggers and pursuing more definitive treatments for VAs, serving as a bridge to procedures such as catheter ablation, surgical interventions, or advanced heart failure treatments.

### Cardiac sympathetic denervation

Initially employed by Estes and Izlar^9^ in 1961 and later by Zipes and colleagues^10^ in 1968 for treating VT, CSD entails the surgical removal of a portion of the lower one-third to one-half of the left or bilateral stellate ganglia and the thoracic ganglia spanning from T2 to T4. The preferred method for conducting this procedure is through video-assisted thoracoscopic surgical techniques, which have contributed to decreased perioperative complications and shorter hospital stays. Surgical CSD has become a potential therapeutic option for individuals who do not respond to more conservative medical interventions and is indicated in cases of VT or VT/fibrillation storms resistant to anti-arrhythmic drugs.

Surgical CSD offers long-term autonomic control by excising the lower portion of the stellate ganglia and the second through fourth thoracic paravertebral ganglia, leading to a decrease in both outgoing and incoming sympathetic nerve signals while preserving some sympathetic innervation. As CSD involves the disruption of preganglionic sympathetic fibers and removal of a segment of stellate and thoracic post-ganglionic neurons, there is no regeneration or reinnervation over time.

### Renal sympathetic denervation

RDN is a catheter-based therapy used for refractory VAs, which reduces whole-body sympathetic activity through the ablation of renal sympathetic nerves. RDN is conducted percutaneously with the assistance of fluoroscopy and electroanatomic mapping guidance. RDN could serve as a supplementary therapy for VT as CSD does not target the catecholamines released by the adrenal gland into the bloodstream and does not decrease overall sympathetic activity systemically. The prospect of a catheter-based intervention targeting autonomic dysregulation linked to structural heart conditions is appealing due to its brief procedure duration, minimal anesthesia requirements, and low associated risks.

## Stereotactic body radiation therapy for VT

Stereotactic body radiation therapy (SBRT) for VT—also referred to as radioablation or cardiac radiosurgery—has emerged as a promising noninvasive modality for patients with refractory VT who have failed or are not candidates for conventional catheter ablation. Unlike catheter-based techniques, SBRT delivers highly focused radiation to the arrhythmogenic substrate identified through noninvasive electroanatomic mapping and advanced cardiac imaging, without requiring vascular access or intracardiac catheterization. This renders the procedure accessible to patients with severe comorbidities who would otherwise be prohibited from invasive procedures.

Early clinical experience, most notably from the ENCORE-VT (“Electrophysiology-guided Noninvasive Cardiac Radioablation for Treatment of Ventricular Tachycardia”) trial^11^ performed at Washington University in St. Louis, demonstrated meaningful acute reductions in VT burden following SBRT in a high-risk patient population with refractory disease. Subsequent registry data from multiple centers have corroborated these initial findings, with several patients experiencing significant and durable reductions in VT episodes and ICD therapies following treatment.

The VT-ART (“Ventricular Tachycardia Ablation through Radiation Therapy”) consortium^12^ represents an ongoing international effort to systematically evaluate SBRT for VT across multiple centers using matched pair analysis to address patient selection bias. This collaborative framework is designed to generate more robust evidence regarding long-term safety and efficacy.

The precise mechanism by which radiation modifies arrhythmogenic tissue remains under investigation. Proposed mechanisms include direct myocardial fibrosis within the targeted substrate, autonomic denervation of the treated region, and remodeling of ion channels and gap junctions critical to arrhythmia propagation. While these mechanistic hypotheses are not mutually exclusive, a complete understanding of radioablation’s electrophysiological effects will be necessary to optimize patient selection, target delineation, and dosing strategies.

Radioablation for VT remains investigational, and long-term safety data—particularly regarding late radiation-related cardiac toxicity—are still being established. Current evidence supports cautious enthusiasm: SBRT may be a meaningful option for carefully selected patients with otherwise refractory VT, but broader adoption awaits results from ongoing prospective trials. Continued advancements in noninvasive mapping, image guidance, and radiation delivery technology will be essential to realizing the full potential of this modality.

## Experimental and investigational modalities

Several energy-based ablation technologies have been explored in preclinical or limited clinical settings for cardiac arrhythmia treatment but currently have no registered or actively enrolling clinical trials for VT ablation. These modalities are best regarded as historically notable and scientifically informative but of limited immediate clinical relevance to VT management. They are summarized briefly below for completeness.

### Laser ablation

Laser ablation employs photon energy interactions with endocardial tissue to induce coagulative necrosis through controlled thermal elevation. In animal studies, a 20-mm linear laser ablation catheter demonstrated the ability to achieve transmural lesions in right ventricular tissue without steam pops or microbubbles, suggesting a degree of procedural safety in preclinical models. However, no human studies have evaluated laser ablation specifically for VT. Its primary clinical application has been in atrial fibrillation, where the HeartLight^®^ X3 endoscopic system (CardioFocus, Marlborough, MA, USA) received United States Food and Drug Administration clearance in 2020 for pulmonary vein isolation. Until evidence specific to VT is generated, laser ablation must be considered an experimental modality for ventricular applications.

### Microwave ablation

MWA generates heat through dipole rotation of water molecules in response to alternating electromagnetic fields, typically operating at 915 or 2450 MHz. While MWA has been evaluated for the surgical treatment of atrial fibrillation—Knaut et al.^13^ reported a 98.5% survival rate in 202 patients with 63% in sinus rhythm at 6 months—its application in VT ablation has not been substantively investigated. MWA has largely fallen out of clinical favor due to concerns about the depth and uniformity of tissue heating and the absence of ongoing device development programs. There is currently no established role for MWA in VT management.

### High-intensity focused ultrasound

HIFU ablation uses focused ultrasonic waves to induce local hyperthermia and cell death through acoustic cavitation and mechanical stress. While early studies demonstrated technical feasibility for pulmonary vein isolation in atrial fibrillation, HIFU trials were suspended following serious safety concerns, particularly the occurrence of esophageal injury and lethal atrio-esophageal fistula formation, even with safety algorithms in place. No human trials to date have evaluated HIFU specifically for VT ablation. Given the unresolved safety profile and absence of active clinical development, HIFU cannot currently be recommended as a viable approach for cardiac arrhythmia ablation.

## Conclusion

This comprehensive review has outlined the principal catheter ablation and neuromodulation techniques employed in the treatment of VT. RFA remains the cornerstone of VT management, with a robust evidence base supported by landmark trials including SMASH-VT and decades of clinical refinement. Innovations including irrigated-tip catheters, high-density electroanatomic mapping, contact force sensing, and needle ablation catheters have progressively expanded the substrates amenable to successful ablation. Cryoablation and PFA offer complementary modalities with distinct safety profiles, and PFA in particular represents a rapidly maturing technology with growing evidence in VT.

RMN has established a meaningful role as an adjunctive technology, while sympathetic denervation techniques provide an important bridge and adjunct for patients with refractory VAs. SBRT represents the most significant emerging noninvasive modality for refractory VT, with early evidence supporting cautious optimism pending longer-term data. Modalities such as laser ablation, MWA, and HIFU, while informative historically, do not currently have active clinical roles in VT management.

Ultimately, optimal VT ablation requires individualized approaches guided by patient characteristics, anatomical substrate, operator expertise, and available technology. Continued innovation—in energy delivery, mapping precision, artificial intelligence integration, and noninvasive targeting—promises to further enhance the efficacy and safety of VT ablation, offering improved outcomes and quality of life for patients with these challenging arrhythmias.

## References

[1] Wellens HJJ, Duren DR, Lie KI (1978). Observations on mechanism of ventricular tachycardia in man. Circulation.

[2] Josephson ME, Horowitz LN, Farshidi A, Kastor JA (1978). Recurrent sustained ventricular tachycardia: 1. Mechanisms. Circulation.

[3] Stevenson WG, Wilber DJ, Natale A (2008). Irrigated radiofrequency catheter ablation guided by electroanatomic mapping for recurrent ventricular tachycardia after myocardial infarction: the multicenter Thermocool Ventricular Tachycardia Ablation Trial. Circulation.

[4] Morady F, Harvey M, Kalbfleisch SJ, el-Atassi R, Calkins H, Langberg JJ (1993). Radiofrequency catheter ablation of ventricular tachycardia in patients with coronary artery disease. Circulation.

[5] Reddy VY, Reynolds MR, Neuzil P (2007). Prophylactic catheter ablation for the prevention of defibrillator therapy. N Engl J Med.

[6] Cáceres J, Werner P, Jazayeri M, Akhtar M, Tchou P (1988). Efficacy of cryosurgery alone for refractory monomorphic sustained ventricular tachycardia due to inferior wall infarction. J Am Coll Cardiol.

[7] Zhang Z, Xiao Y, Wang C (2024). Pulsed field ablation: A promising approach for ventricular tachycardia ablation. Int J Cardiol.

[8] Lerich R, Fontaine R (1973). Local anesthesia of the stellate ganglion. Surv Anesthesiol.

[9] Estes EH, Izlar HL (1961). Recurrent ventricular tachycardia. A case successfully treated by bilateral cardiac sympathectomy. Am J Med.

[10] Zipes DP, Festoff B, Schaal SF, Cox C, Sealy WC, Wallace AG (1968). Treatment of ventricular arrhythmia by permanent atrial pacemaker and cardiac sympathectomy. Ann Intern Med.

[11] Robinson CG, Samson PP, Moore KMS (2019). Phase I/II trial of electrophysiology-guided noninvasive cardiac radioablation for ventricular tachycardia. Circulation.

[12] Cellini F, Narducci ML, Pavone C (2023). Ventricular tachycardia ablation through radiation therapy (VT-ART) consortium: Concept description of an observational multicentric trial via matched pair analysis. Front Cardiovasc Med.

[13] Knaut M, Tugtekin SM, Jung F, Matschke K (2004). Microwave ablation for the surgical treatment of permanent atrial fibrillation—a single centre experience. Eur J Cardiothorac Surg.

